# Global Vaccine Equity to End the COVID-19 Pandemic: A Canadian Perspective and Call to Action

**DOI:** 10.3389/ijph.2022.1604729

**Published:** 2022-01-27

**Authors:** Michael Clarke, Shehzad Ali, Michael Silverman, Saverio Stranges

**Affiliations:** ^1^ Interfaculty Program in Public Health, Western University, London, ON, Canada; ^2^ Department of Epidemiology and Biostatistics, Western University, London, ON, Canada; ^3^ The World Health Organization Collaborating Centre for Knowledge Translation and Health Technology Assessment in Health Equity, Bruyère Research Institute, Ottawa, ON, Canada; ^4^ Division of Infectious Diseases, Western University, London, ON, Canada; ^5^ Department of Family Medicine, Western University, London, ON, Canada, ON, Canada; ^6^ Department of Medicine, Western University, London, ON, Canada; ^7^ Department of Population Health, Luxembourg Institute of Health, Strassen, Luxembourg

**Keywords:** COVID-19, vaccine distribution, equity, patent challenges, Canada

While much of the world awaits a better understanding of the threat posed by the omicron variant of COVID-19, the rest of the world wonders if they will ever get vaccinated. There is overwhelming evidence on widening inequalities in the health and economic burden from the current pandemic, as low-resources settings and marginalized population subgroups have paid the highest toll [1].

Globally, the World Health Assembly is working to define equity in a legally binding treaty to address pandemic preparedness [2]—in the context of the current COVID-19 pandemic, this treaty would provide the legal context for fair and just distribution of COVID-19 vaccines globally.

The essential first step towards equity in COVID-19 vaccination programs is access to vaccines. Although actual global supply of vaccines may not be a limiting factor in achieving equity, the unequal (and inequitable) distribution of vaccines is clearly a formidable barrier for low- and middle-income countries. WHO’s COVAX program has delivered only one-seventh of the commitments from high-income countries and Canada, itself, has drawn almost one million doses from the program while delivering only 3.2 million of the 40 million doses promised [3]. There have been calls for a waiver from intellectual property rights from the current COVID-19 vaccine manufacturers, which would allow production of patented vaccines by generic pharmaceutical companies at lower cost [4–6].

Formal mechanisms for suspending patent protection for medicines in the event of a national or global emergency including issuing an IP waiver is through the exceptions clause in the Trade-Related Aspects of Intellectual Property Rights (TRIPS) agreement under the so-called Doha declaration [7] in response to national threats to public health in low-income countries. A separate mechanism allows for the issuing of compulsory licenses for the generic manufacture of patented medicines [8]. Canada was the first country to issue a compulsory licence in this case to Apotex Inc., Toronto, ON, Canada, for the manufacture and distribution of TriAvir, an anti-HIV drug, in response to a request from Rwanda.

The legislation that enabled Canada to issue a compulsory license was the Canada Access to Medicines Regime (CAMR) that was passed in 2006. The Act was dubbed “Jean Chretien’s Pledge to Africa” after the Prime Minister at the time. The CAMR process was eventually described as “unworkable” and criticized [9] due to a heavy burden of bureaucratic red tape. However, deliveries of TriAvir were made to Rwanda in 2008 and 2009; however, CAMR has never since been implemented.

In 2012, Bill C-398, designed to streamline the CAMR process by removing several limiting conditions in the original CAMR legislation, was introduced into the House of Commons and was defeated by the majority Conservative government at the time. Bill C-398 was originally supported by the Liberals, New Democrats and the Bloc Quebecois and therefore should be supported by the current Liberal minority government. Indeed, the Prime Minister has recently declared that Canada is, “... not interfering or blocking (*patent waivers*). Canada is very much working to find a solution that works for everyone.” [10]. We propose that the appropriate long-term mechanism to achieve this is a re-introduction and passage of Bill C-398, or a version thereof, by the Canadian government.

The current COVID-19 pandemic and the obviously skewed and unjust global distribution of vaccines represent solid grounds for employing a compulsory license or waiver for the generic manufacture of vaccines. The legal and legislative processes are in place for Canada to do the right thing. Donations of vaccines from high-income countries may alleviate inequity in the short term [2]. However, the establishment of locally owned vaccine manufacturing capacity in low- and middle- income regions lessening their dependence on donor funding is a more sustainable option in the longer term.

Compulsory licenses and IP waivers are not a panacea for addressing equity; however, at this point in the COVID-19 pandemic, it is a necessary step. Most low- and middle-income countries have the experience and immunization programs in place that can be leveraged to tackle global pandemics. However, these programs are chronically under-funded and have persistent inequalities. This is where Canada, and the Global North, also need to think beyond vaccine supply.

To truly prepare for the next pandemic, we call upon all countries, particularly Canada, with capacity or plans for vaccine manufacture to respect the principles of the Doha declaration and enact legislation to grant compulsory licenses or IP waivers for the rapid scale-up of vaccine production and distribution in preparation for the next inevitable global pandemic.
